# New Sesquiterpenoids from the Fermented Broth of *Termitomyces albuminosus* and Their Anti-Acetylcholinesterase Activity

**DOI:** 10.3390/molecules24162980

**Published:** 2019-08-16

**Authors:** Wei Li, Qian Liu, Shimian Cheng, Shanren Li, Yongbiao Zheng

**Affiliations:** 1 Engineering Research Centre of Industrial Microbiology, Ministry of Education, College of Life Sciences, Fujian Normal University, Fuzhou 350117, China; 2 Provincial University Key Laboratory of Cellular Stress Response and Metabolic Regulation, College of Life Sciences, Fujian Normal University, Fuzhou 350117, China; 3 Fujian Provincial University Engineering Research Center of Industrial Biocatalysis, College of Chemistry and Material Sciences, Fujian Normal University, Fuzhou 350117, China

**Keywords:** *Termitomyces albuminosus*, selinane, sesquiterpenoids, anti-acetylcholinesterase, microbial fermentation

## Abstract

*Termitomyces albuminosus* is the symbiotic edible mushroom of termites and cannot be artificially cultivated at present. In the project of exploring its pharmaceutical metabolites by microbial fermentation, four new selinane type sesquiterpenoids—teucdiol C (**1**), D (**2**), E (**3**), and F (**4**), together with two known sesquiterpenoids teucdiol B (**5**) and epi-guaidiol A (**6**)—were obtained from its fermented broth of *T. albuminosus*. Their structures were elucidated by the analysis of NMR data, HR Q-TOF MS spectral data, CD, IR, UV, and single crystal X-ray diffraction. *Epi*-guaidiol A showed obvious anti-acetylcholinesterase activity in a dose-dependent manner. The experimental results displayed that *T. albuminosus* possess the pharmaceutical potential for Alzheimer’s disease, and it was an effective way to dig new pharmaceutical agent of *T. albuminosus* with the microbial fermentation technique.

## 1. Introduction

*Termitomyces albuminosus* (Berk.) Heim is the symbiotic edible mushroom of termites [1]. The fruiting bodies of *T. albuminosus* are rich in nutritional and medicinal constituents. Many compounds with medicinal potentials have been obtained from its dried fruiting bodies, such as novel cerebrosides termitomycesphins A–H with significant neuritogenic activity [2,3,4] and cerebroside A with the potent neuroprotection activity [5]. *T. albuminosus* has also displayed antioxidant capacity and high content phenolic ingredients [6]. However, *T. albuminosus* must grow at a termitarium and cannot be cultivated artificially at present. In previous reports, the microbial fermentation technology has been proven to be an effective method to utilize the natural resources of *T. albuminosus*. It has been reported that the mycelia of *T. albuminosus* obtained by microbial fermentation contained an extraordinarily high amount of α-aminobutyric acid (2.56 g/kg [7]), possessed a highly intense umami taste [8], and had antioxidant properties [9]. Saponins and polysaccharides from the dry matter of culture broth of *T. albuminosus* possessed the analgesic and anti-inflammatory activities [10]. In this paper, we mainly focus on investigating the pharmaceutical metabolites of *T. albuminosus* by the method of microbial fermentation and describe the structure elucidation and bioactivities of these compounds.

## 2. Results

### 2.1. Purification and Characterization of Sesquiterpenoids

The edible mushroom *T. albuminosus* was cultured in flasks each containing 100 mL of potato dextrose media with a total volume of 25.9 L. These flasks were incubated for 30 days at 28 °C with a shaking speed of 210 rpm. The fermented broth whose mycelia were removed by filtration were extracted with ethyl acetate. Then ethyl acetate phase was dried over anhydrous sodium sulfate and concentrated under reduced pressure to afford 3.28 g of a crude organic extract. The crude extract was successively subject to column chromatography over reverse phase-18 silica gel, Sephadex LH-20, and silica gel to afford six compounds (**1**–**6**).

Based upon the detailed analysis of NMR data, including ^1^H, ^13^C, DEPT (Distortionless Enhancement by Polarization Transfer), HSQC, HMBC and ^1^H-^1^H COSY spectra (Table 1 and Table 2 and Figures S1–S14), compounds **1**–**6** were identified as selinane type sesquiterpenoids (Figure 1). These sesquiterpenoids contain a similar decahydronaphthalene carbon skeleton. The major difference of these compounds is the isopropyl groups linked at C-7.

**Figure 1:**
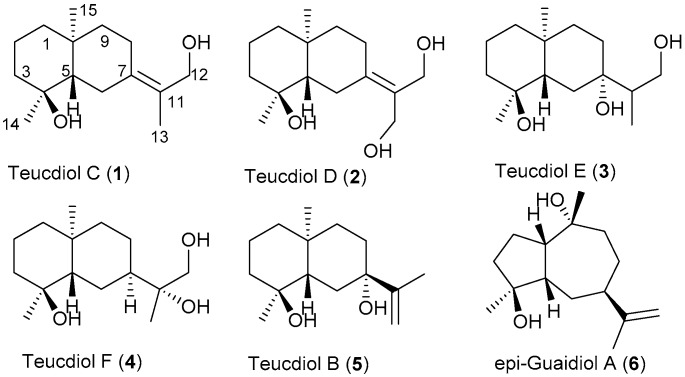
The chemistry structures of compounds **1**–**6**.

**Figure 2:**
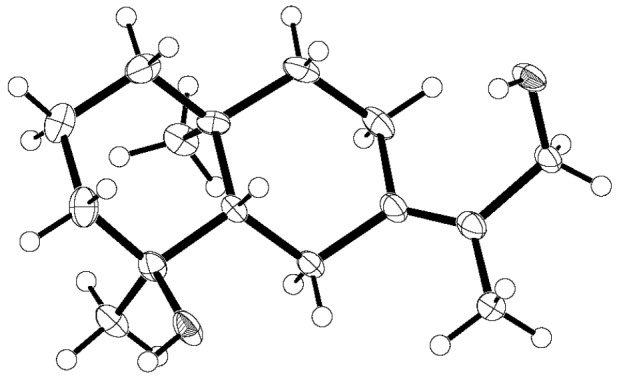
The crystal form of compound **1**.

Compound **1** was obtained as an amorphous colorless substance with an optical value of [*α*]${}_{D}^{25}$−10.3 (c 0.1, methanol) and a maximum UV absorption of 210 nm in methanol. The molecular formula of compound **1** was determined to be C_15_H_26_O_2_ based on the high-resolution quadrupole time-of-flight mass spectrometry (HR Q-TOF MS) peak at *m/z*: 261.1823 (calculated for C_15_H_26_O_2_Na, 261.1830) and ^1^H and ^13^C-NMR data (Table 1 and Table 2 and Figures S1 and S2). In the IR spectra, the prominent absorption indicated the presence of a –OH group (3386 cm^−1^). NMR data (^1^H, ^13^C, DEPT) revealed resonances for three methyls, seven methylenes (including one –CH_2_OH group (δC 63.4)), one methine (δC 56.5), and four quaternary carbons, including one oxygenated carbon (δC 73.1) and two *sp*^2^ carbons (δC 125.9; δC 138.2). Thus, compound **1** must be a bicyclic sesquiterpenoid containing one double bond for three degrees of unsaturation based upon its molecular formula and NMR data. The obvious HMBC correlations from H_3_-14 to C-3/C-4/C-5 and from H_3_-15 to C-1/C-5/C-9/C-10, as well as ^1^H-^1^H COSY cross-peaks between both H-1 and H-2 and H-2 and H-3 allowed for the establishment of one cyclic moiety of compound **1**. Another cyclic moiety of **1** was deduced from HMBC correlations from H_3_-13 to C-11/C-12/C-7, from H_2_-12 to C-11/C-13/C-7, from H_2_-6 to C-4/C-7/C-8/C-10/C-11, and from H_2_-9 to C-1/C-5/C-7/C-8/C-10/C-15, as well as ^1^H-^1^H COSY cross-peaks between H-5 and H-6, H-8 and H-9. Thus, the basic structure of compound **1** could be established (Figure 1). The configuration of compound **1** was deduced by the Nuclear Overhauser Effect Spectrometry (NOESY) experiments. The cross-peaks between H-8α and H_3_-15, H-2α and H_3_-15, H-9α and H_3_-15, H-2α and H_3_-14, and H-6α and H-8α in the NOESY spectrum indicated the α-orientation of these protons. The other NOEs between H-5 and H-1β, H-5 and H-3β, and H-5 and H-6β allowed for the assignment of the β-orientation of these protons. The stereochemistry structure of compound **1** was confirmed by X-ray diffraction of the single crystal obtained from the aqueous methanol (Figure 2). Crystallographic data (CCDC 1938575) for compound **1**: C_15_H_26_O_2_, white crystal, triclinic, space group P1, *a* = 7.9272(10) Å, *b* = 9.0784(12) Å, *c* = 11.0248(16) Å, α = 83.510(11)°, *β* = 71.243(12)°, *γ* = 68.555(12)°, *V* = 699.26(18) Å^3^, *Z* = 3, *Dc* = 1.227 g·cm^−3^, F(000) = 273, and Flack parameter = −0.3(3). According to the above data, the stereochemistry structure of compound **1** was deduced, and it was named teucdiol C (Figure 1).

Compound **2** was obtained as an amorphous colorless substance with an optical value of [*α*]${}_{D}^{25}$−18.4 (c 0.1, methanol) and a maximum UV absorption of 216 nm in methanol. The molecular formula of compound **2** was determined to be C_15_H_26_O_3_ based on the HR Q-TOF MS peak at *m/z*: 277.1776 (calculated for C_15_H_26_O_3_Na, 277.1780) and ^1^H and ^13^C NMR data (Table 1 and Table 2 and Figures S3 and S4). In the IR spectra, the prominent absorption indicated the presence of a –OH group (3385 cm^−1^). NMR data (^1^H, ^13^C, DEPT) revealed resonances for two methyls, eight methylenes (including two –CH_2_OH groups (δC 60.29 and δC 60.31)), one methine (δC 56.7), and four quaternary carbons, including one oxygenated carbon (δC 73.2) and two *sp*^2^ carbons (δC 130.3; δC 144.1). The analysis of 1D- and 2D-NMR spectral data (^1^H, ^13^C, DEPT, HSQC, HMBC, ^1^H-^1^H COSY) displayed that compound **2** as a hydroxyl derivative of compound **1** at the position of C-13. Besides, compounds **1** and **2** have the same configuration based upon the same negative optical value and the same positive cotton effect showed in the circular dichroism spectra (Figures S5 and S6). The protons’ orientation of compound **2** was further confirmed by the NOESY experiments. NOEs between H_3_-15 and H-8α, H-3α and H_3_-15, H-2α and H_3_-15, H-3α and H_3_-14, and H-6α and H_3_-14 in the NOESY spectrum indicated the α-orientation of these protons. The other NOEs between H-5 and H-1β as well as H-5 and H-6β allowed for the establishment of the β-orientation of these protons. Thus, the stereochemistry of compound **2** was established, and it was named teucdiol D (Figure 1). Compound **2** showed weak activity against *Escherichia coli* at the concentration of 0.98 mM in our filed patent [11].

Compound **3** was obtained as an amorphous colorless substance with an optical value of [*α*]${}_{D}^{25}$+13.6 (c 0.1, methanol) and a maximum UV absorption of 201 nm in methanol. The molecular formula of compound **3** was determined to be C_15_H_28_O_3_ based on the HR Q-TOF MS peak at *m/z*: 279.1926 (calculated for C_15_H_28_O_3_Na, 279.1936) and ^1^H and ^13^C-NMR data (Table 1 and Table 2 and Figures S7 and S8). In the IR spectra, the prominent absorption indicated the presence of a hydroxyl group (3420 cm^−1^). NMR data (^1^H, ^13^C, DEPT) revealed resonances for three methyls, seven methylenes (including one –CH_2_OH group (δC 65.3)), two methines (δC 51.9; δC 37.1), and three quaternary carbons, including two oxygenated carbon (δC 72.8 and δC 76.7). Compound **3** must be bicyclic sesquiterpenoid for the two degrees of unsaturation required by the molecular formula and the decahydronaphthalene skeleton. The isopropyl group (C11–C12–C13) linked at C-7 was hydroxyl in the position of C-12. Thus, the planar structure of **3** was established. The configuration of compound **3** was further confirmed by the NOESY experiment. These NOEs of H_3_-15α with H-2α, H_3_-15α with H-9α, H_3_-15α with H-8α, H_3_-15α with H-1α, H_3_-15α with H-6α, H_3_-14α with H-3α, and H_3_-14α with H-2α indicated the α-orientation of these protons in compound **3**. The other NOEs between H-5 and H-8β, H-5 and H-11, H-1β and H-9β, and H-11 and H-9β, allowed for the β-orientation of these protons in compound **3**. Then, the stereochemistry of compound **3** was deduced, and it was named teucdiol E (Figure 1).

Compound **4** was obtained as an amorphous colorless substance with an optical value of [*α*]${}_{D}^{25}$+8.7 (c 0.1, methanol) and a maximum UV absorption of 200 nm in methanol. In the IR spectra, the prominent absorption indicated the presence of a –OH group (3416 cm^−1^). The molecular formula of compound **4** was determined to be C_15_H_28_O_3_ based on the HR Q-TOF MS peak at *m/z*: 279.1930 (calculated for C_15_H_28_O_3_Na, 279.1936) and ^1^H and ^13^C-NMR data (Table 1 and Table 2 and Figures S9 and S10). The above data indicated that compounds **3** and **4** were isomers with similar carbon chemical shifts. However, a detailed analysis revealed that the OH group, which was linked at C-7 in compound **3**, was connected at C-11 in compound **4**, based upon these evidences of the singlet peak of H_3_-13, the downfield chemical shift of C-13 (δ 23.6), the obvious cross-peak between H-6 and H-7, and HMBC correlations from H-7 to C-11/C-9/C-5/C-6/C-13. Thus, the basic structure of compound **4** was yielded. NOEs of H_3_-15α with H-8α, H_3_-15α with H-9α, H_3_-15α with H-2α, H_3_-15α with H-1α, H_3_-14α with H-3α, H_3_-14α with H-9α, H_3_-14α with H-2α, H-7 with H-6α, and H-7 with H-8α indicated the α-orientation of these protons in compound **4**. The other NOEs between H-5 and H-1β, H-5 and H-9β, H-5 and H-2β, H-5 and H-8β, and H-5 and H-3β allowed for the β-orientation of these protons in compound **4**. Moreover, the obvious cross-peaks of H_3_-13 and H-5, H_3_-13 and H-6β, H_2_-12 and H-6β, and H_2_-12 and H-8β indicated the β-orientation of the methyl and the hydroxymethyl groups. According to the above data, compound **4** was shown to possess the same basic structure of (−)-(11*R*)-eudesm-4α,11,12-triol which was a reduction product by LiAlH_4_ of α-epoxykudtdiol isolated from *Jasonia glutinosa* [12]. However, with compare to the sinistral optical value of (−)-(11*R*)-eudesm-4α,11,12-triol, compound **4** had the dextral optical value. So, the configuration of compound **4** was deduced from these data. Compound **4** was isolated as a natural product for the first time, and it was named teucdiol F (Figure 1).

Compound **5** was obtained as an amorphous colorless substance with an optical value of [*α*]${}_{D}^{25}$+0.06 (c 0.1, methanol) and a maximum UV absorption of 201 nm in methanol. The molecular formula of compound **5** was determined to be C_15_H_26_O_2_ based on the HR Q-TOF MS peak at *m/z*: 261.1831 (calculated for C_15_H_26_O_2_Na, 261.1830) and ^1^H and ^13^C-NMR data (Table 1 and Table 2 and Figures S11 and S12). Through comparison of their NMR data of compound **5** and the known configurational isomers teucdiol A and B [13,14], compound **5** could be identified as teucdiol B with the α-orientation of the hydroxyl group at C-7, based upon the evidence of the downfield chemical shit at C-5 (δC 52.2 for compound **5**, δC 51.1 for teucdiol B, and δC 48.8 for teucdiol A) (Figure 1).

Compound **6** was obtained as an amorphous colorless substance with a sinistral optical value of [*α*]${}_{D}^{25}$−0.005 (c 0.1, methanol) and a maximum UV absorption of 201 nm in methanol. The molecular formula of compound **6** was determined to be C_15_H_26_O_2_ based on the HR Q-TOF MS peak at *m/z*: 261.1834 (calculated for C_15_H_26_O_2_Na, 261.1752) and ^1^H and ^13^C-NMR data (Table 1 and Table 2 and Figures S13 and S14). Compound **6** could be identified as *epi*-guaidiol A [15,16,17] compared with the dextral optical value of guaidiol [18] (Figure 1).

### 2.2. Anti-Acetylcholinesterase Activities of Sesquiterpenoids

Ellman’s assay was used to measure the anti-acetylcholinesterase activity of these sesquiterpenoids [19,20]. Except for compounds **1**–**5**, the experimental data displayed that epi-guaidiol A (compound **6**) showed obvious anti-acetylcholinesterase activity in a dose-dependent manner (Table 3). Recently, some sesquiterpenoids from food were reported to possess anti-acetylcholinesterase activity. A new seco-illudoid sesquiterpene—pterosinone from *Pteridium aquilinum*—showed mild acetylcholinesterase and butyrylcholinesterase inhibitory activity with IC_50_ value (Half inhibition concentration) of 87.7 and 72.9 mM respectively [21]. α-Isocubebenol isolated from *Schisandra chinensis* fruit could repress acetylcholinesterase activity and alleviate scopolamine-induced cognitive impairment [22]. The sesquiterpenes in *Vernonia oligocephala* extracts showed acetylcholinesterase inhibitory potential [23]. The major chemical constituent of essential oil from *Lavandula pedunculata* are monoterpenes, and sesquiterpenes and showed the most active against acetylcholinesterase [24]. As mentioned above, sesquiterpenoids with anti-acetylcholinesterase activity could be a potential natural therapeutic agent for Alzheimer’s disease. However, the inhibition mechanistic and action model of the above inhibitors, which were screened by the limited methods, were unclear [25]. More data including the dissociation constant and kinetics parameters are needed for unveiling their reaction mechanism [26]. The isolated compound (**6**, *epi*-guaidiol A) in this paper is also awaited in unveiling its inhibition mechanism against acetylcholinesterase before the application of the pharmaceutical function of mushroom *T. albuminosus* in the future.

## 3. Materials and Methods

### 3.1. General Experimental Procedures

NMR spectra were recorded in Bruker ARX 500 spectrometer (Bruker BioSpin Group, Zurich, Switzerland) operating at 500/125 MHz, in ppm relative to Me_4_Si as internal reference; *J* in Hz. UV spectra were measured on a Shimadzu UV-2600 spectrophotometer (Tokyo, Japan) in nm(λmax). IR spectra were recorded on a Bruker Tensor-27 FT-IR spectrophotometer (Ettlingen, Germany) with KBr cells in cm^−1^. Optical rotations were obtained on a Jasco P-1020 automatic polarimeter (Tokyo, Japan). HR Q-TOF MS spectra were recorded on an Agillent 6520 mass spectrometer (Agilent Technologies, Santa Clara, CA, USA) in *m/z*. Circular dichroism (CD)spectra were measured on a Chirascan Plus spectroscope (Applied photophysics, Leatherhead, Surrey, UK). X-ray single diffraction was performed on an Oxford Gemini S Ultra diffractometer (Rigaku, Oxford, UK). Column chromatography was performed with silica gel (Qingdao Marine Chemical Company, Qingdao, China), reverse phase octadecyl-silica (Merck, Darmstadt, Germany), and Sephadex LH20 (Amersham Biosciences, Piscataway, NJ, USA). Thin layer chromatography was performed on the precoated silica gel plates (GF254, Qingdao Marine Chemical Company, Qingdao, China). Organic solvents used were from Sino-pharm Chemical Reagent Co., Ltd. (Shanghai, China).

### 3.2. Fungus Material

The strain *T. albuminosus* was supplied by Xie Bao-gui (Fungal Research Centre, Fujian Agriculture and Forestry University, Fuzhou, China). The strain was deposited in College of Life Sciences, Fujian Normal University and was deposited in the China Centre for Type Culture Collection (CCTCC M 2016262).

### 3.3. Fermentation and Preparation of Extracts

*T. albuminosus* was cultured in flasks, each containing 100 mL of potato dextrose media with a total volume of 25.9 L. These flasks were incubated for 30 days at 28 °C with a shaking speed of 210 rpm. The fermented broth, whose mycelia were removed by filtration, was extracted with ethyl acetate. The ethyl acetate phase was dried over anhydrous sodium sulfate and concentrated under a reduced pressure to afford 3.28 g of a crude organic extract.

### 3.4. Isolation and Purification of Sesquiterpenoids ***1**–**6***

The crude extract was subjected to medium pressure liquid chromatography (MPLC)over RP-18 silica gel (170 g) using a stepwise gradient of 30%, 50%, 70%, and 100% (*v/v*) MeOH in water and to afford Fr.1 (68.3 mg), Fr.2 (100.4 mg), and Fr.3 (101.0 mg) obtained from 50% MeOH in water and Fr. 4 (289.4 mg) obtained from 70% MeOH in water. Then fractions Fr.1–4 were subjected to a Sephadex LH-20 column (100 g) eluted with MeOH to afford Fr.11 (44.0 mg), Fr.21 (53.7 mg), Fr.31 (21.1 mg), and Fr.41 (206.8 mg). Fr.11 was further subjected to the Sephadex LH-20 column (130 g) eluted with acetone to afford Fr.111 (3.9 mg) and Fr.112 (7.8 mg). Fr.111 and Fr.112 were subjected to silica gel (1.0 g) chromatography using a CHCl_3_–MeOH solvent gradient to yield compound **2** (2.8 mg). Fr.21 (53.7 mg) was further subjected to MPLC over RP-18 silica gel (30 g) using a stepwise gradient of 40%, 42%, and 44% (*v/v*) MeOH in water to afford Fr.211 (13.9 mg) obtained from 44% MeOH in water. Then, Fr.211 was subjected to silica gel (1.3 g) chromatography using a CHCl_3_–MeOH solvent gradient to yield compound **3** (11.9 mg). Fr.31 was subjected to silica gel (2 g) chromatography using a CHCl_3_–MeOH solvent gradient to yield compound **4** (16.2 mg). Fr.41 (206.8 mg) was further subjected to the Sephadex LH-20 column (130 g) eluted with acetone to afford Fr.411 (11.0 mg), Fr.412 (12.0 mg), and Fr.413 (6.6 mg). Then sub-fractions Fr.411, Fr.412, and Fr.413 were subjected to silica gel (1.3, 1.4, and 0.8 g, respectively) chromatography using a CHCl_3_–MeOH solvent gradient to yield compound **1** (6.3 mg), compound **5** (6.4 mg), and compound **6** (2.2 mg) respectively.

### 3.5. Colorimetric Determination of Acetylcholinesterase Activities

Ellman’s assay was used to measure acetylcholinesterase activity in 96-well microtiter plates in a final reaction volume of 200 μL. First, 50 μL of a 0.05 M sodium phosphate buffer (pH = 7.0) and 20 μL of 5 mg/mL compounds dissolved in 25% ethanol were added in each well. Then, 10 μL of 1 μg/mL EelAchE (Sigma-Aldrich, Inc., product number C2888) dissolved in a 0.02 M phosphate buffer (pH = 7.0) containing BSA (Beijing Dingguo Changsheng Biotechnology Company, FA016-5G, Beijing, China) was added in each well and put at 4 °C for 20 min. Secondly, 20 μL of 1.05 mM acetylthiocholine (Sigma-Aldrich, Inc., product number BCBR6567V) and 100 μL of 1.5 mM 5,5′-dithio-bis-nitrobenzoicacid (Shanghai Aladdin Bio-Chem Technology Company, J1530009, Shanghai, China) were added to each well before being mixed and reacted at 37 °C for 20 min. Thirdly, each well was subjected to colorimetric determination at 412 nm by a microtiter plate reader (Synergy HT, BioTek Instruments, Winooski, VT, USA). 20 μL of 0.11 mg/mL huperzine A (Aladdin, F1517037) was set as the positive control group. 20 μL of 25% ethanol in water was set the negative control. Percentage inhibition was calculated using the following formula:

Inhibition rate (%) = ((A_0_ − A_1_)/A_0_) × 100

where A_0_ was the absorbance of the negative control and A_1_ was the absorbance of the samples. Tests were carried out in triplicate.

### 3.6. X-ray Single Crystal Diffraction for Compound ***1***

X-ray single diffraction was performed on an Oxford Gemini S Ultra single crystal diffractor (Rigaku, Oxford, UK). A suitable crystal was selected and subjected to λ(Cu−kα) = 1.54184 Å at 273.15 K. The structure was determined using the direct method and refined with full-matrix least squares calculations on *F*^2^ using olex2, and 8570 reflections were measured (8.4702 ≤ 2θ ≤ 132.4376); of these, 4398 unique reflections (*R_int_* = 0.0572) were used in all calculations. The final *wR*_2_ was 0.1646 (all data) and *R*_1_ was 0.0536 (*I* ≥ 2*σ* (*I*)). Crystallographic data for compound **1** was deposited with the Cambridge Crystallographic Data Center (CCDC 1938575 for compound **1**). Crystallographic data (CCDC 1938575) for compound **1**: C_15_H_26_O_2_, white crystal, triclinic, space group P1, *a* = 7.9272(10) Å, *b* = 9.0784(12) Å, *c* = 11.0248(16) Å, α = 83.510(11)°, *β* = 71.243(12)°, *γ* = 68.555(12)°, *V* = 699.26(18) Å^3^, *Z* = 3, *Dc* = 1.227 g·cm^−3^, F(000) = 273, and Flack parameter = −0.3(3).

## 4. Conclusions

In our lab, microbial fermentation was used to explore the metabolites of some edible and medicinal mushroom. Many new pharmaceutical agents have been discovered by this culture method [27,28,29,30,31]. It was concluded that this is also an effective way to dig for new pharmaceutical agents of *T. albuminosus* with the microbial fermentation technique. We also revealed that mushroom *T. albuminosus* possesses pharmaceutical potential for Alzheimer’s disease.

## Figures and Tables

**Table 1:** ^1^H-NMR spectral data of **1**–**6**.

No.	1	2	3	4	5	6
1α	1.44 (o, 1H) ^1^	1.45 (o, 1H)	1.42 (o, 1H)	1.37 (o, 1H)	1.74 (o,1H)	2.04 (m, 1H)
1β	1.11 (o, 1H)	1.10 (o, 1H)	1.10 (o, 1H)	1.13 (dd, *J* = 12.7, 4.7 Hz, 1H)	1.35–1.29 (o,1H)	
2α	1.60 (o, 1H)	1.56 (o, 1H)	1.58 (o, 1H)	1.53 (o, 1H)	2.31 (dt, *J* = 12.9, 2.5 Hz, 1H)	1.67–1.69 (o, 2H)
2β	1.65 (o, 1H)	1.63 (o, 1H)	1.62 (o, 1H)	1.40 (o, 1H)	1.40 (o, 1H)	
3α	1.79 (o, 1H)	1.77 (o, 1H)	1.77 (m, 1H)	1.76 (o, 1H)	1.74 (o, 1H)	1.93 (m, 1H)
3β	1.40 (o, 1H)	1.42 (m, 1H)	1.38 (m, 1H)	1.44 (o, 1H)	1.35–1.29 (o, 1H)	1.64 (o, 1H)
5	1.26 (dd, *J* = 13.0, 2.8 Hz, 1H)	1.27 (o, 1H)	1.30 (o, 1H)	1.62 (o, 1H)	1.23 (dd, *J* = 13.1, 2.2 Hz, 1H)	2.58 (m, 1H)
6α	1.71 (o, 1H)	1.80 (o, 1H)	1.34 (o, 1H)	1.46 (o, 1H	1.60–1.54 (o, 2H)	1.76 (o, 1H)
6β	2.91 (d, *J* = 13.0 Hz, 1H)	2.99 (m, 1H)	2.24 (dd, *J* = 11.7, 2.8 Hz, 1H)	1.97 (m, 1H))		1.51 (o, 1H)
7				1.91 (m, 1H)		2.15 (td, *J* = 10.9, 4.1 Hz, 1H)
8α	2.04 (m, 1H)	2.09 (m, 1H)	1.47 (m, 1H)	1.65 (o, 1H)	1.67 (m, 1H)	1.63 (o, 1H)
8β	2.67 (m, 1H)	2.68 (m, 1H)	1.84 (m, 1H)	1.81 (o, 1H)		1.40 (o, 1H)
9α	1.49 (o, 1H)	1.52 (o, 1H)	1.26 (o, 1H)	1.56 (o, 1H)	1.04 (dd, *J* = 12.9, 4.8 Hz, 1H)	1.83 (o, 1H)
9β	1.20 (m, 1H)	1.24 (o, 1H)	1.21 (td, *J* = 13.8, 3.4 Hz, 1H)	1.20 (m, 1H)		1.56 (o, 1H)
11			2.02 (m, 1H)			
12	4.10 (s, 2H)	4.20 (s, 2H)	3.76 (m, 2H)	3.48 (d, *J* = 3.9 Hz, 2H)	5.10 (s, 1H)	4.66 (m, 1H)
					5.01 (s, 1H)	4.59 (m, 1H)
13	1.80 (s, 3H)	4.24 (s, 2H)	1.04 (d, *J* = 7.0 Hz, 3H)	1.22 (s, 3H)	1.82 (s, 3H)	1.71 (s, 3H)
14α	1.14 (s, 1H)	1.12 (s, 3H)	1.09 (s, 3H)	1.08 (s, 3H)	1.09 (s, 3H)	1.14 (s, 3H)
15α	1.04 (s, 1H)	1.04 (s, 3H)	0.98 (s, 3H)	0.94 (s, 3H)	0.99 (s, 3H)	1.21 (s, 3H)

^1^ Recorded at 500 MHz in MeOD; λ in ppm, *J* in Hz.

**Table 2:** ^13^C-NMR spectral data of **1**–**6**.

No.	1	2	3	4	5	6
1	42.3t ^1^	42.2t	42.0t	43.3t	43.7t	53.6d
2	21.2t	21.3t	21.3t	21.4t	32.3t	40.7t
3	44.0t	44.2t	44.0t	44.5t	43.7t	42.7t
4	73.1s	73.20s	72.8s	73.5s	72.8s	75.7s
5	56.5d	56.7d	51.9d	50.5d	52.2d	52.9d
6	26.4t	26.2t	32.9t	22.1t	21.2t	31.7t
7	138.2s	130.3s	76.7s	38.7d	76.0s	48.6d
8	26.5t	27.0t	33.3t	21.7t	33.3t	33.0t
9	47.1t	46.9t	42.7t	43.5t	42.2t	26.47t
10	36.1s	36.2s	35.9s	35.5s	36.0s	82.0s
11	125.9s	144.1s	37.1d	77.4s	148.1s	153.7s
12	63.4t	60.29t	65.3t	69.8t	114.0t	108.5t
13	16.7q	60.31t	12.1q	23.6q	19.2q	20.5q
14	22.3q	22.3q	22.5q	22.2q	22.7q	24.0q
15	18.8q	18.9q	19.3q	19.7q	19.5q	26.52q

^1^ Recorded at 500 MHz in MeOD; *λ* in ppm, *J* in Hz.

**Table 3:** The inhibition rate of compound **6** against acetylcholinesterase activity.

Concentration of Compound 6 (mM)	Inhibition Rate (%)
2.10	56.2 ± 0.8 ^1^
1.57	53.4 ± 4.0
1.05	44.5 ± 3.6
0.52	33.6 ± 3.8
Positive control	94.6 ± 1.5
Vehicle	6.40 ± 1.9

^1^ The value is the average for three replicate and standard deviation.

## Supplementary Materials

Supplementary data associated with this article are available online. ^1^H and ^13^C-NMR spectra for all compounds, circular dichroism spectroscopy for compounds **1** and **2**, and X-ray crystallographic data for compound **1** are presented.
