# Supplementary material for: New Sesquiterpenoids from the Fermented Broth of Termitomyces albuminosus and Their Anti-Acetylcholinesterase Activity
Source: Molecules. 2019 Aug 16;24(16):2980. doi: 10.3390/molecules24162980 (PMC6719985; doi:10.3390/molecules24162980)
Supplement: Supplementary file 1 [file molecules-24-02980-s001.pdf]

Supplementary Materials

New Sesquiterpenoids from the Fermented Broth of *Termitomyces albuminosus* and the Anti-Acetylcholinesterase Activity

Wei Li [1,2], Qian Liu [1,2,] Shimian Cheng [1,2], Shanren Li [1,2] and Yongbiao Zheng [1,2,3,*]

[1] Engineering Research Centre of Industrial Microbiology, Ministry of Education; College of Life Sciences, Fujian Normal University, Fuzhou 350117, China
[2] Provincial University Key Laboratory of Cellular Stress Response and Metabolic Regulation, College of Life Sciences, Fujian Normal University, Fuzhou 350117, China
[3] Fujian Provincial University Engineering Research Center of Industrial Biocatalysis, College of Chemistry and Material Sciences, Fujian Normal University, Fuzhou 350117, China
* Correspondence: yongbiaozheng@fjnu.edu.cn

The List of Contents

No.	Content	Page
1	Figure S1 ^{1}H NMR spectra of compound **1** (in MeOD)	2
2	Figure S2 ^{13}C NMR spectra of compound **1** (in MeOD)	2
3	Figure S3 ^{1}H NMR spectra of compound **2** (in MeOD)	3
4	Figure S4 ^{13}C NMR spectra of compound **2** (in MeOD)	3
5	Figure S5 The circular dichroism spectra of **1** (in methanol)	4
6	Figure S6 The circular dichroism spectra of **2** (in methanol)	4
7	Figure S7 ^{1}H NMR spectra of compound **3** (in MeOD)	5
8	Figure S8 ^{13}C NMR spectra of compound **3** (in MeOD)	5
9	Figure S9 ^{1}H NMR spectra of compound **4** (in MeOD)	6
10	Figure S10 ^{13}C NMR spectra of compound **4** (in MeOD)	6
11	Figure S11 ^{1}H NMR spectra of compound **5** (in MeOD)	7
12	Figure S12 ^{13}C NMR spectra of compound **5** (in MeOD)	7
13	Figure S13 ^{1}H NMR spectra of compound **6** (in MeOD)	8
14	Figure S14 ^{13}C NMR spectra of compound **6** (in MeOD)	8

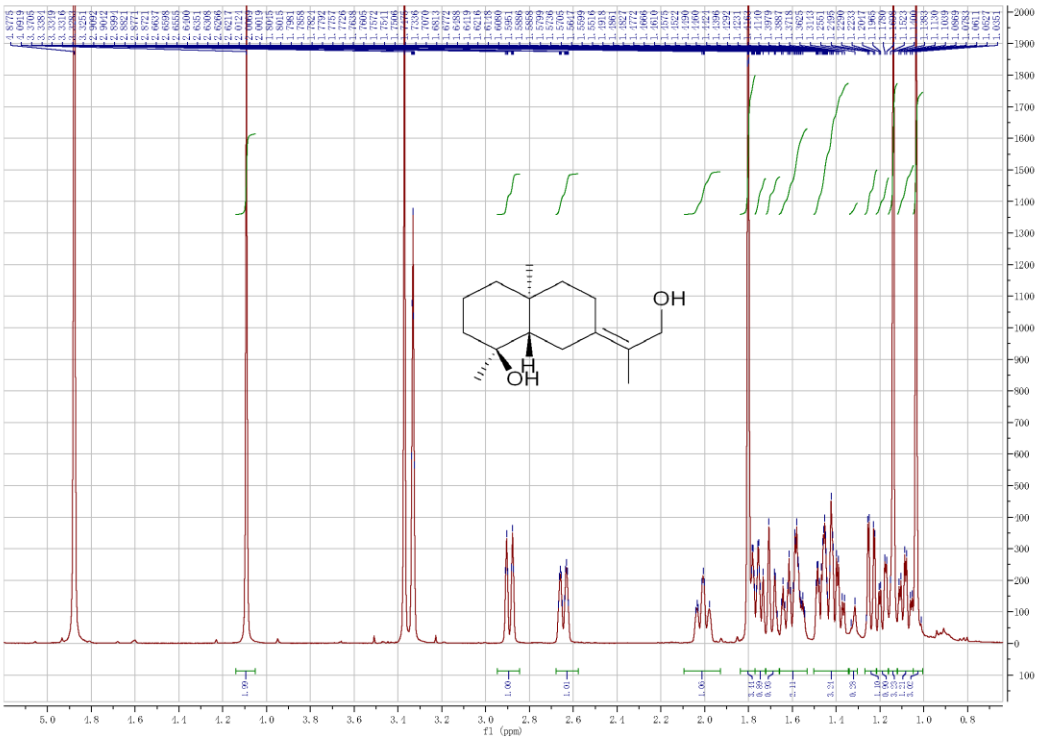

Figure S1. ^{1}H-NMR spectra of compound **1** (in MeOD).

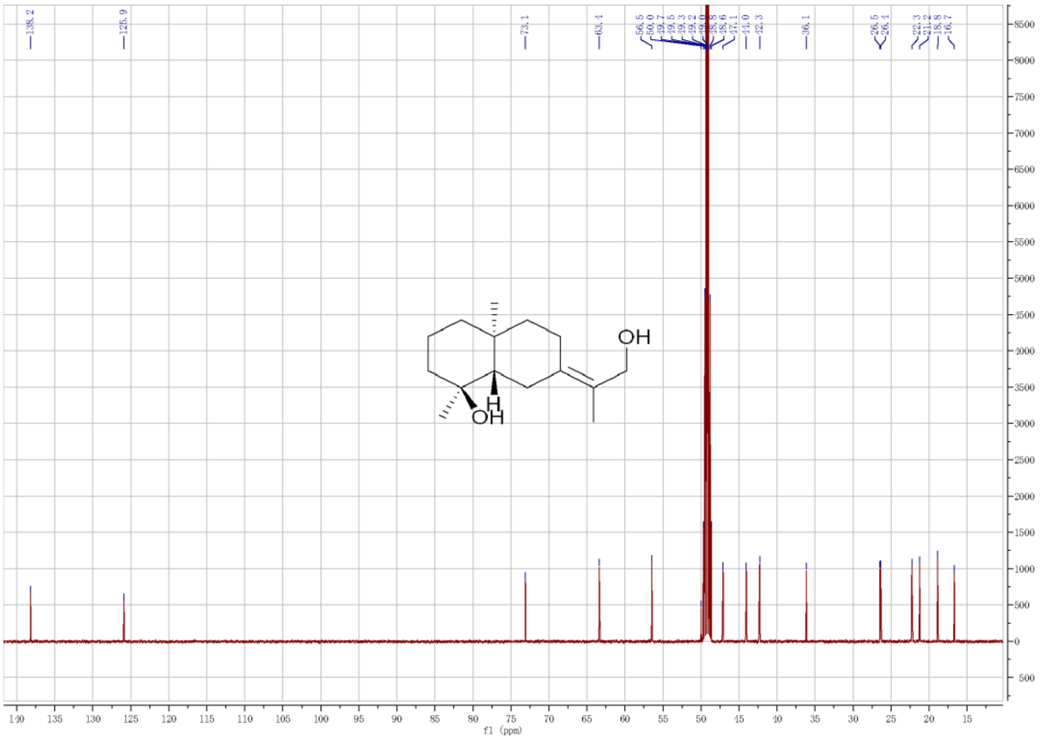

Figure S2. ^{13}C-NMR spectra of compound **1** (in MeOD).

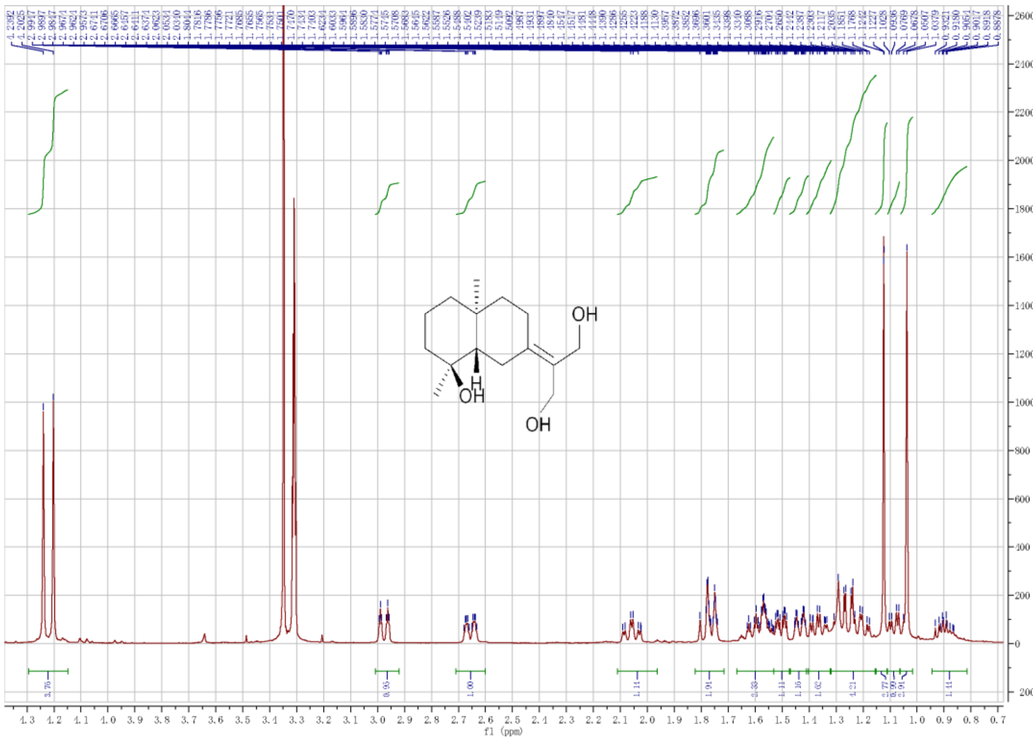

Figure S3. ^{1}H-NMR spectra of compound **2** (in MeOD).

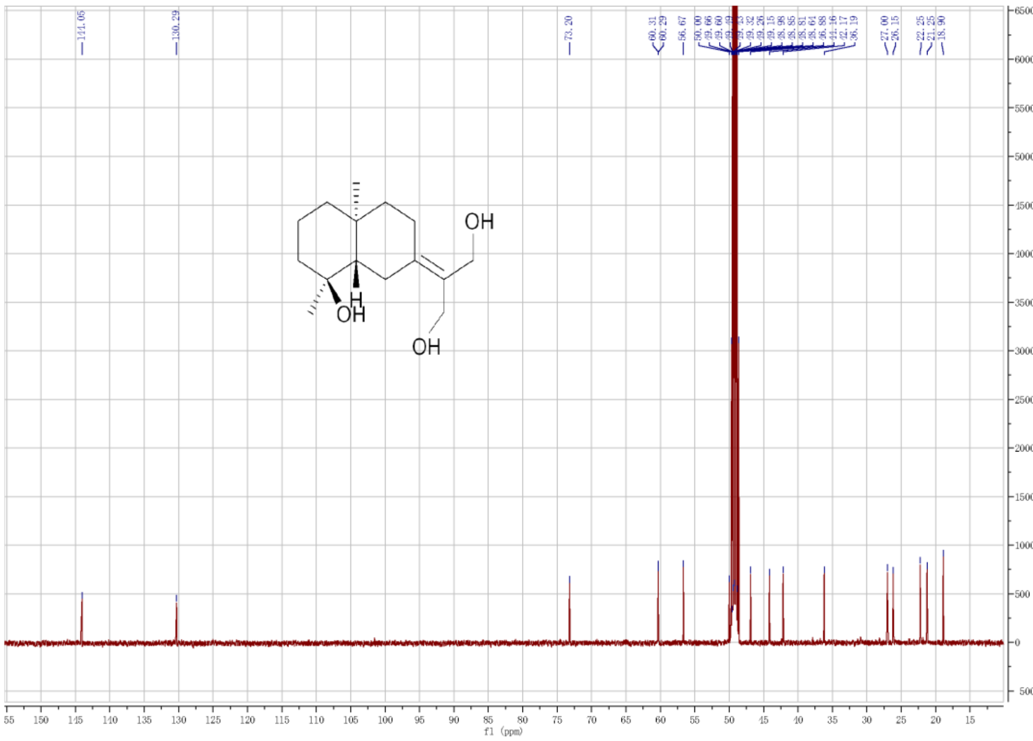

Figure S4. ^{13}C-NMR spectra of compound **2** (in MeOD).

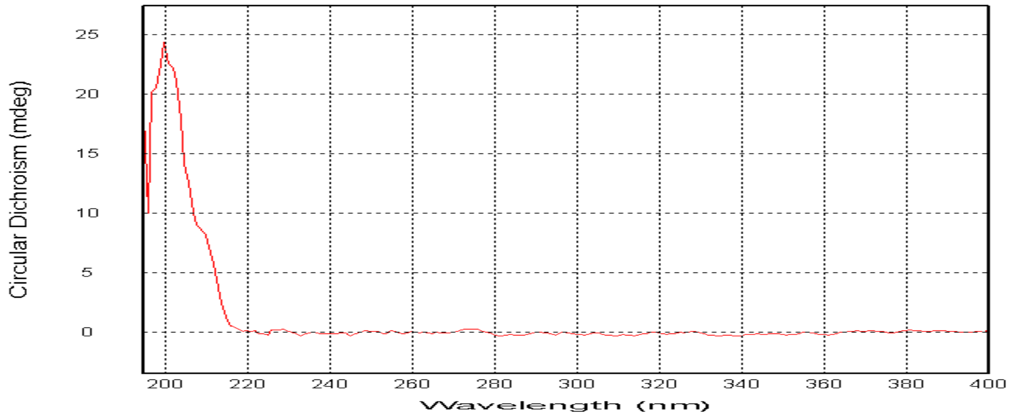

Figure S5. The circular dichroism spectra of **1** (in methanol).

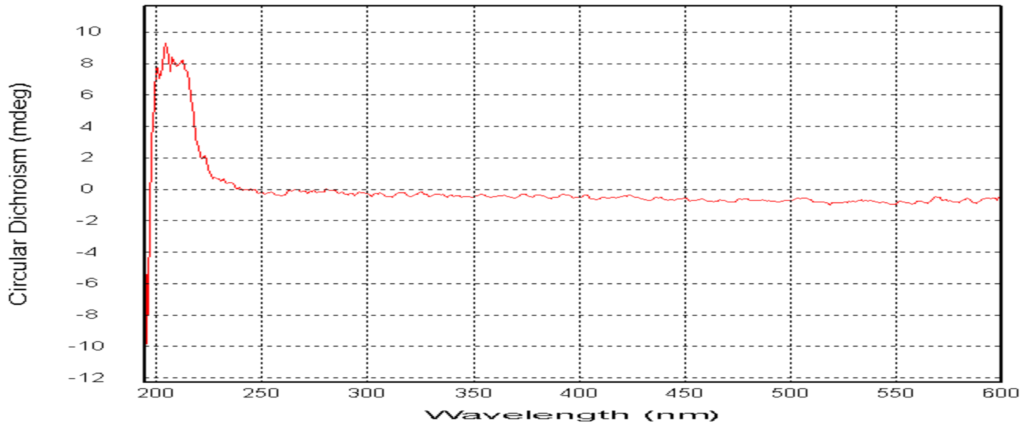

Figure S6. The circular dichroism spectra of **2** (in methanol).

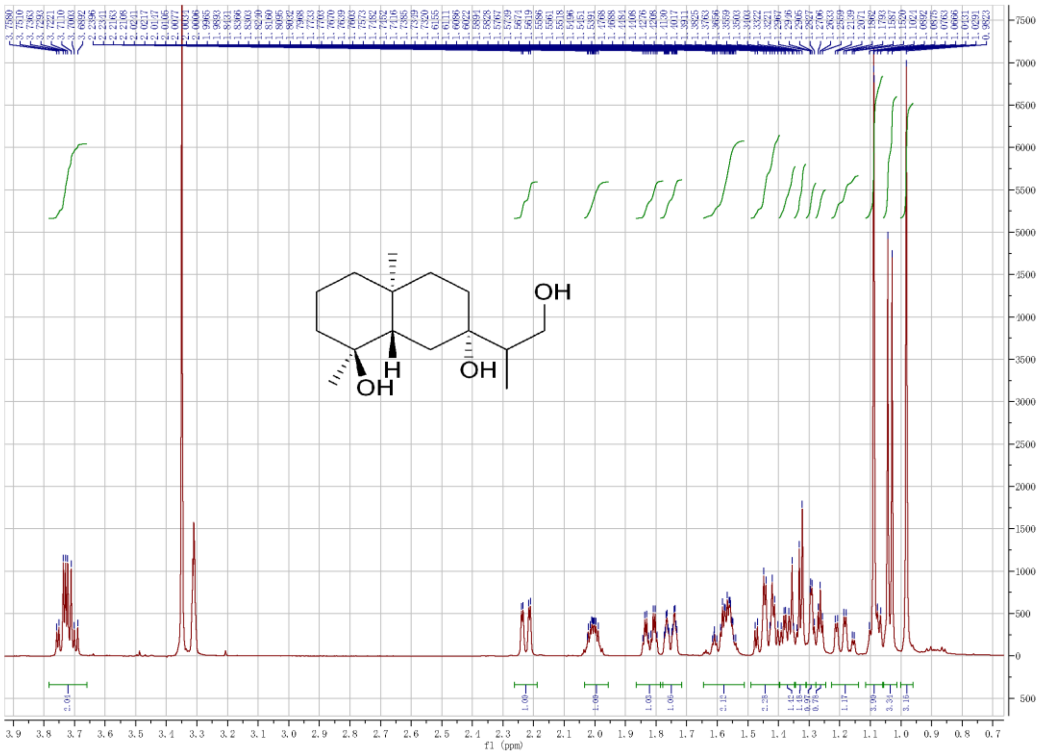

Figure S7. ^{1}H-NMR spectra of compound **3** (in MeOD).

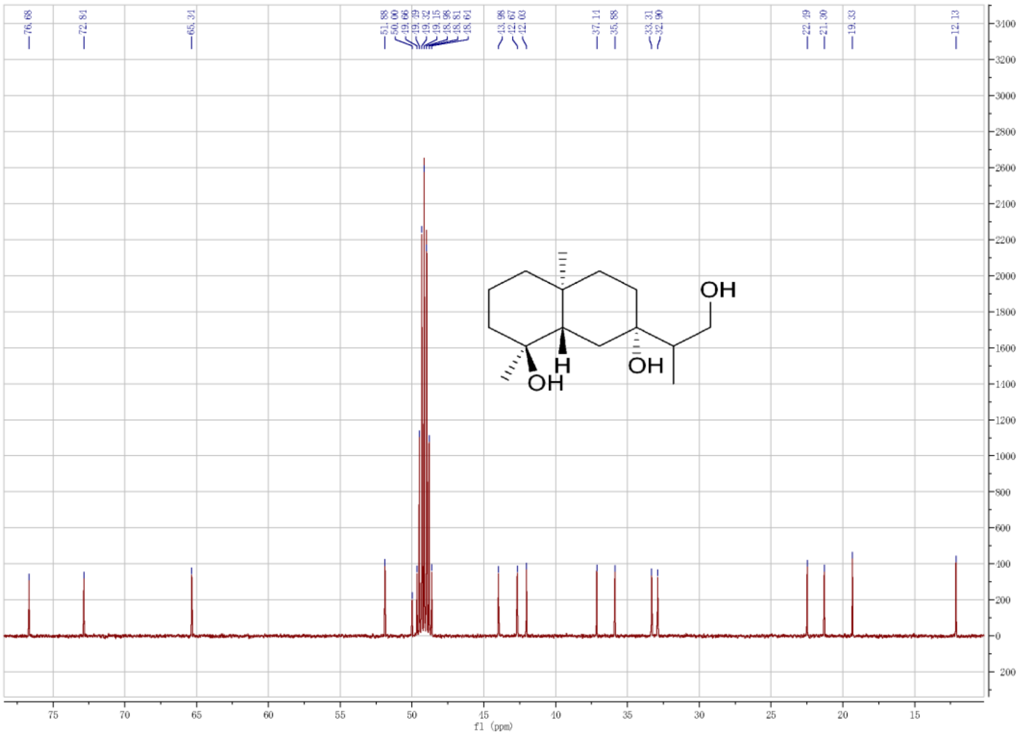

Figure S8. ^{13}C-NMR spectra of compound **3** (in MeOD).

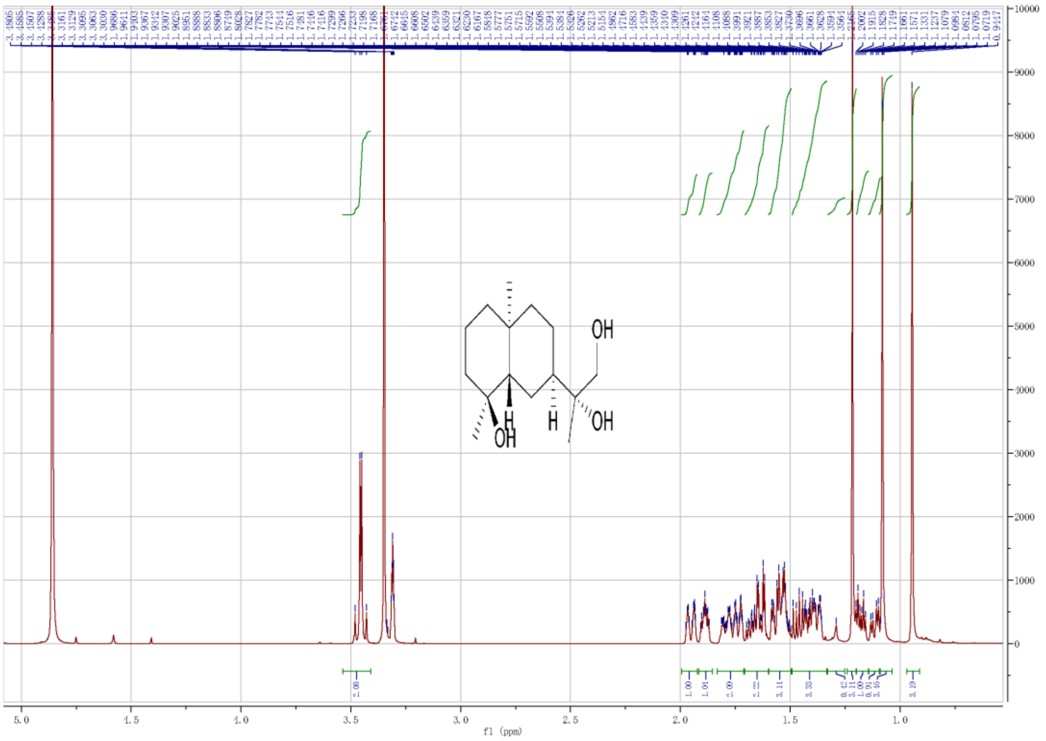

Figure S9. ^{1}H-NMR spectra of compound **4** (in MeOD).

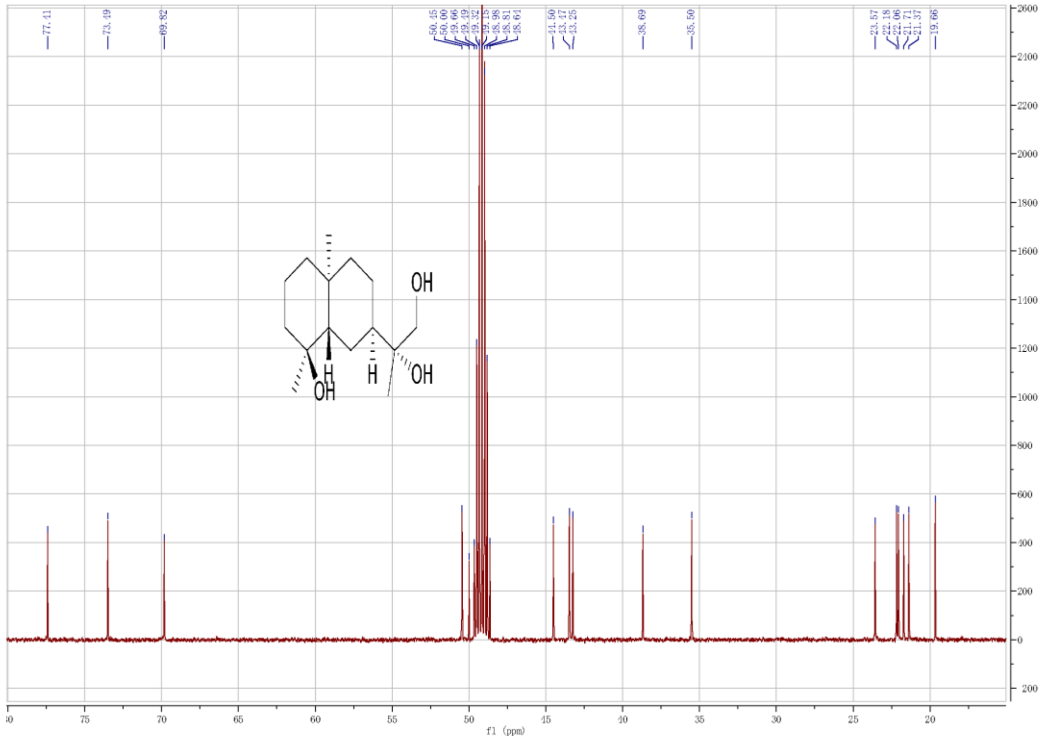

Figure S10. ^{13}C-NMR spectra of compound **4** (in MeOD).

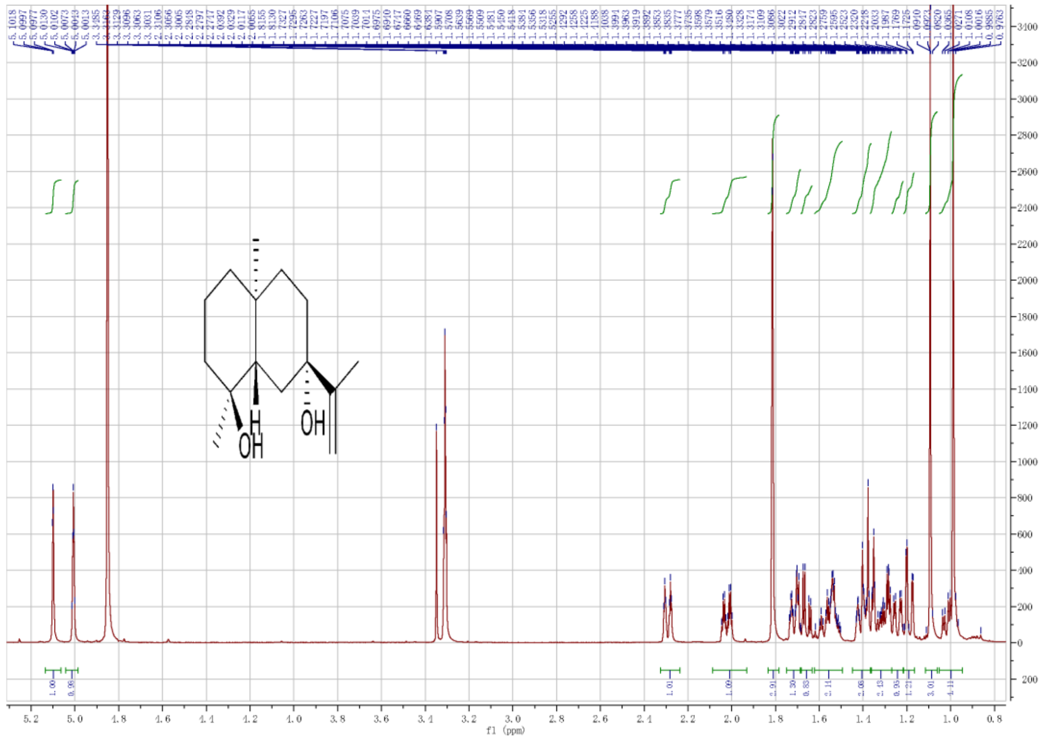

Figure S11. ^{1}H-NMR spectra of compound **5** (in MeOD).

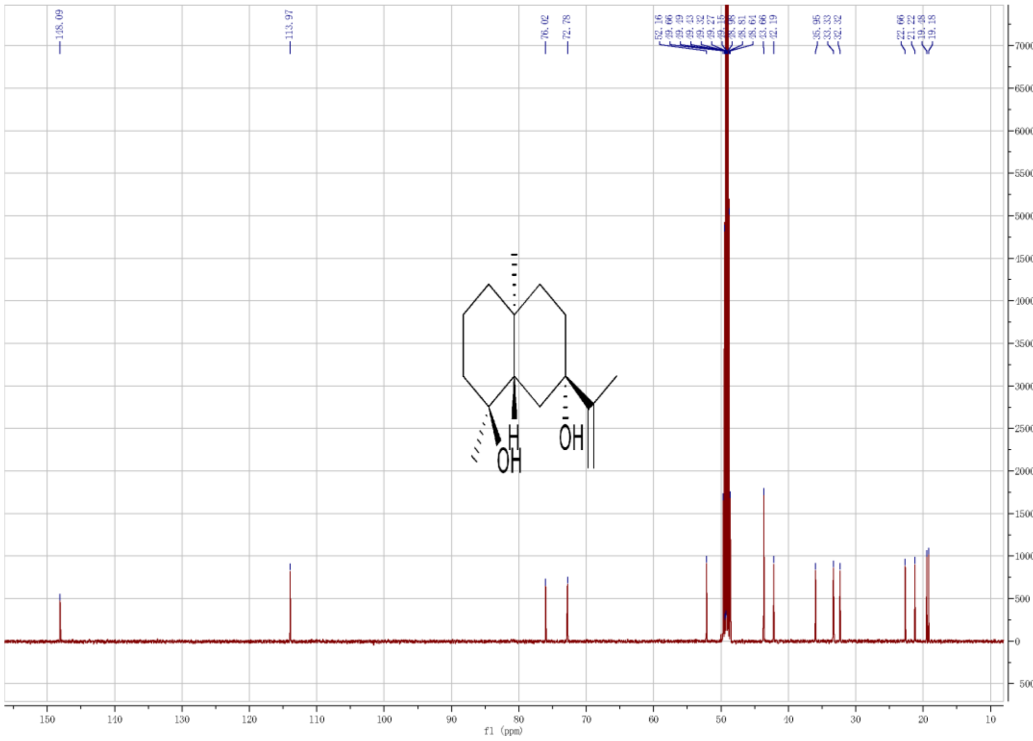

Figure S12. ^{13}C-NMR spectra of compound **5** (in MeOD).

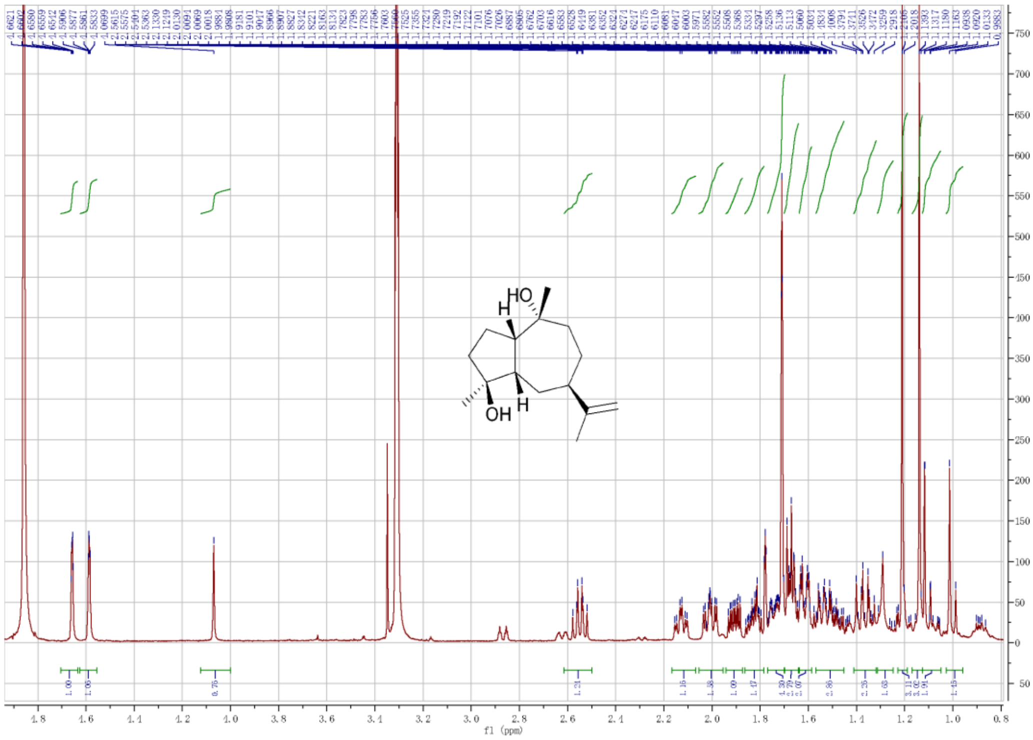

Figure S13. ^{1}H-NMR spectra of compound **6** (in MeOD).

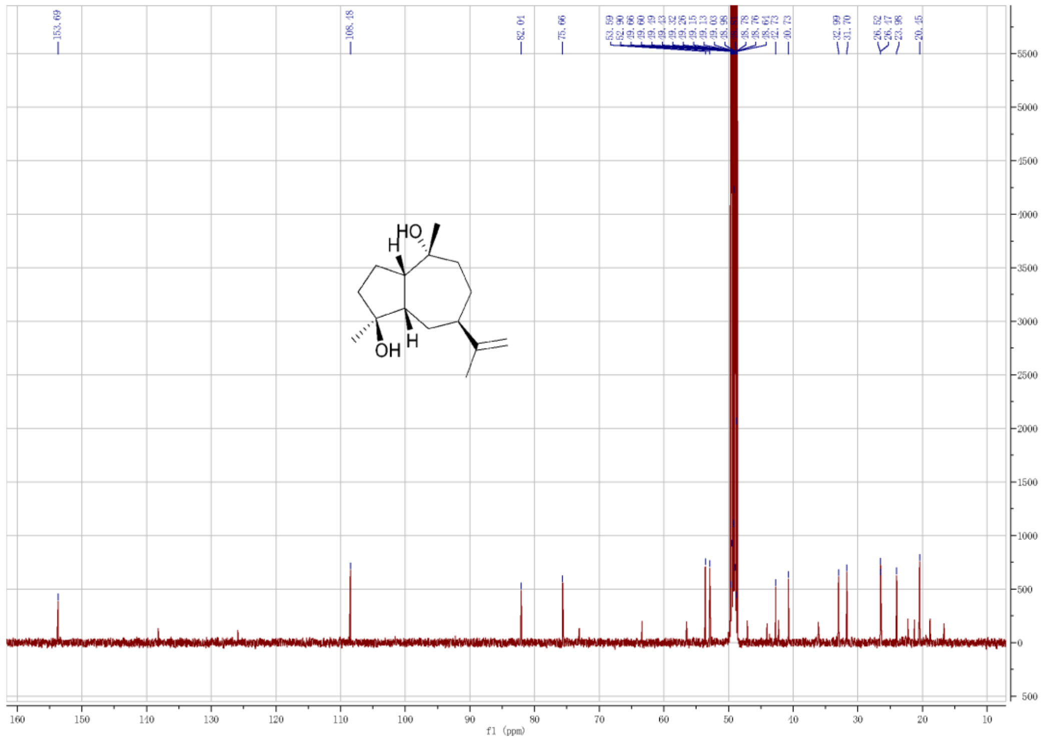

Figure S14. ^{13}C-NMR spectra of compound **6** (in MeOD).
